# Supplementary material for: Nanostructured Glass-Ceramic Materials from Glass Waste with Antimicrobial Activity
Source: Molecules. 2024 Jul 6;29(13):3212. doi: 10.3390/molecules29133212 (PMC11243445; doi:10.3390/molecules29133212)
Supplement: Supplementary file 1 [file molecules-29-03212-s001.zip › molecules-3052644-supplementary.pdf]

Communication

Nanostructured glass-ceramic materials from glass waste with antimicrobial activity

Juliani P. Caland[1], João Baptista[2], Gabrielle Caroline Peiter[2], Kelen M. F. Rossi de Aguiar[2], Horácio Coelho Júnior[3], João P. Sinnecker[3], Jorlandio F. Felix [1,*], and Ricardo Schneider [2,*]

1 Núcleo de Física Aplicada, Instituto de Física, Brasília, Universidade de Brasília-UnB, Brasília 70910-900, DF, Brazil; julianicaland96@hotmail.com

2 Group of Polymers and Nanostructures, Universidade Tecnológica Federal do Paraná - UTFPR, Toledo, 85902-490, PR, Brazil

3 Brazilian Center for Physics Research, Rio de Janeiro 22290-180, RJ, Brazil; horaciocoelhojunior@gmail.com (H.C.-J.)

* Correspondence: jorlandio@unb.br (J.F.F.); rschneider@utfpr.edu.br (R.S.)

Table S 1. Elemental composition of waste glass.

Compound	Amber	Flint
	%	
Na_2O	15.7	16.0
MgO	2.0	2.6
Al_2O_3	1.8	0.8
SiO_2	70.4	70.5
CaO	9.6	9.8
Fe_2O_3	0.5	0.4

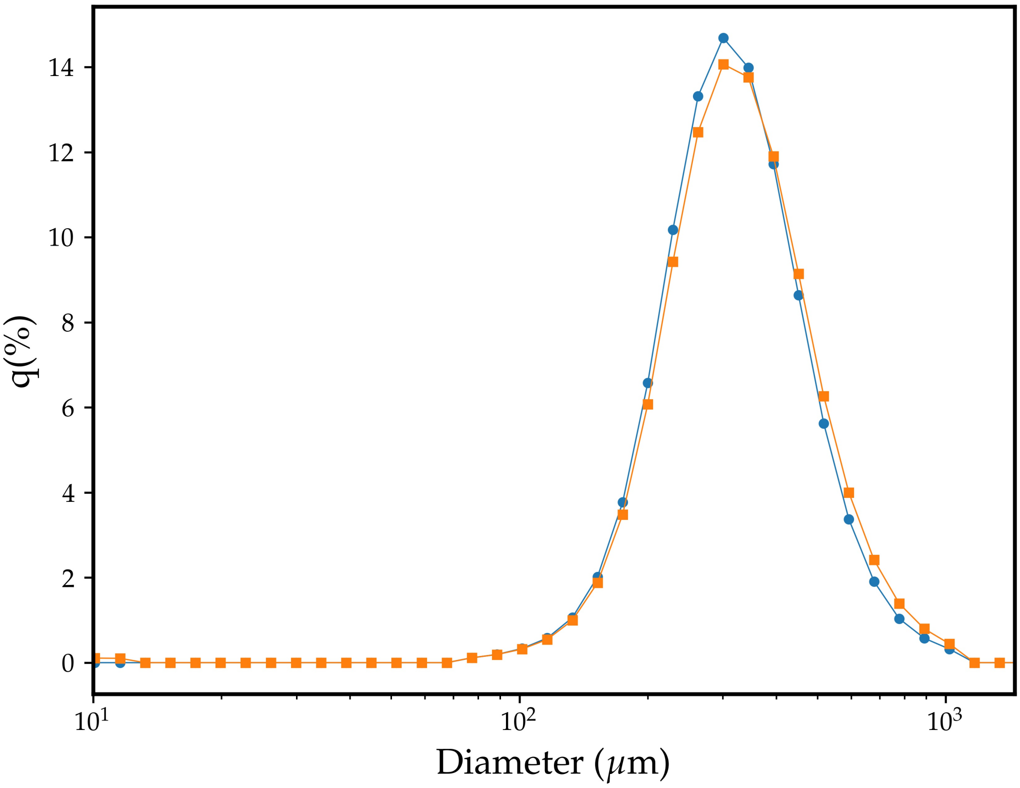

Figure S 1. Particle size distribution for amber waste glass before sintering according to the sieve powder granulometry (Mesh) [48,100). Measurements were performed in duplicate. Mean size ≈ 322.6 μm and geometric standard deviation of 1.5 μm.

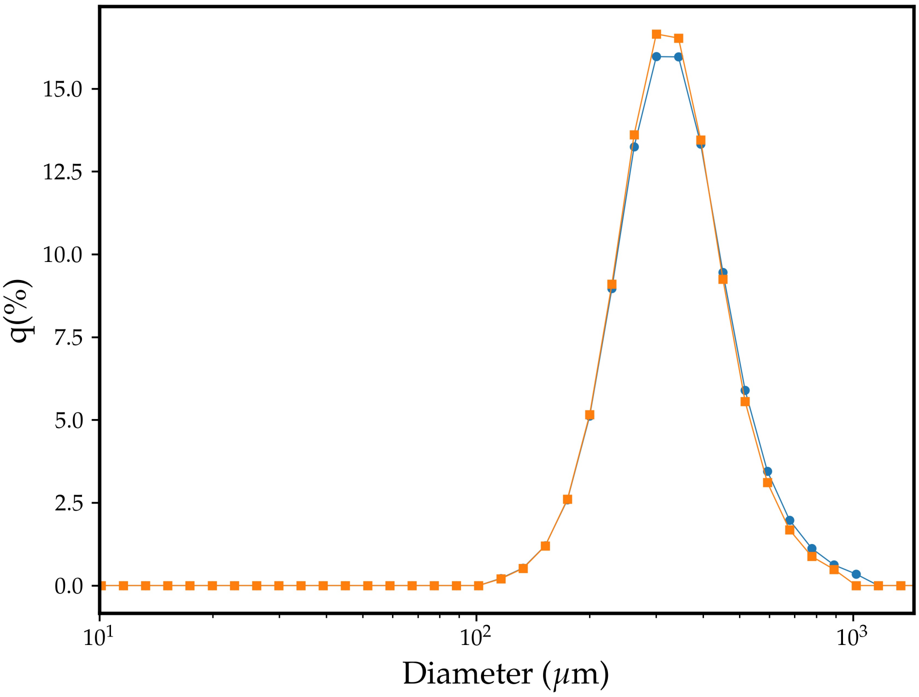

Figure S 2. Particle size distribution for flint waste glass before sintering according to the sieve powder granulometry (Mesh) [48,100). Measurements were performed in duplicate. Mean size ≈ 326.5 μm and geometric standard deviation of 1.4 μm.

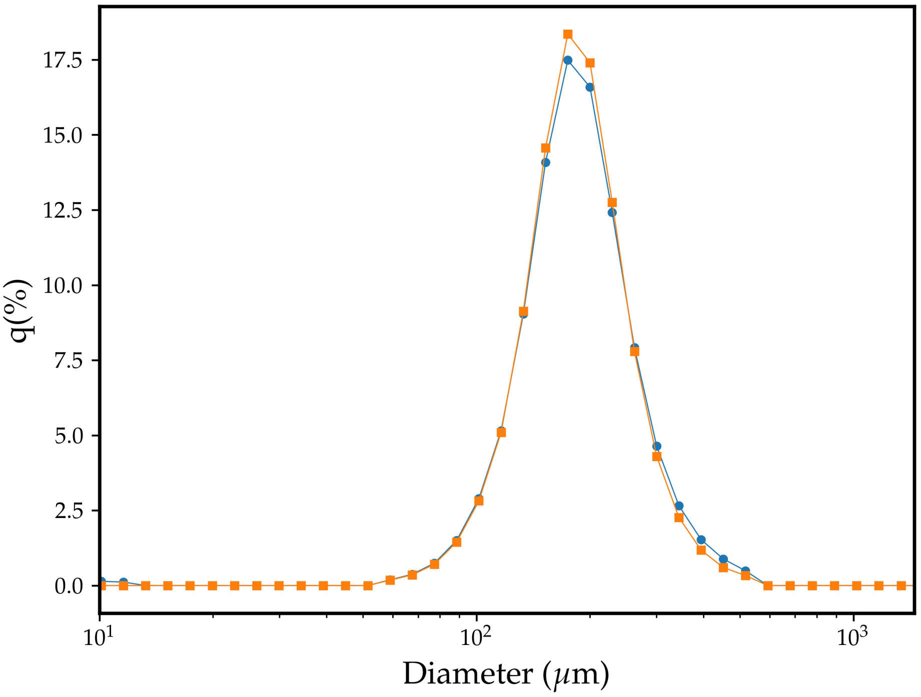

Figure S 3. Particle size distribution for amber waste glass before sintering according to the sieve powder granulometry (Mesh) [100,200). Measurements were performed in duplicate. Mean size $\approx$ 179.9 μm and geometric standard deviation of 1.6 μm.

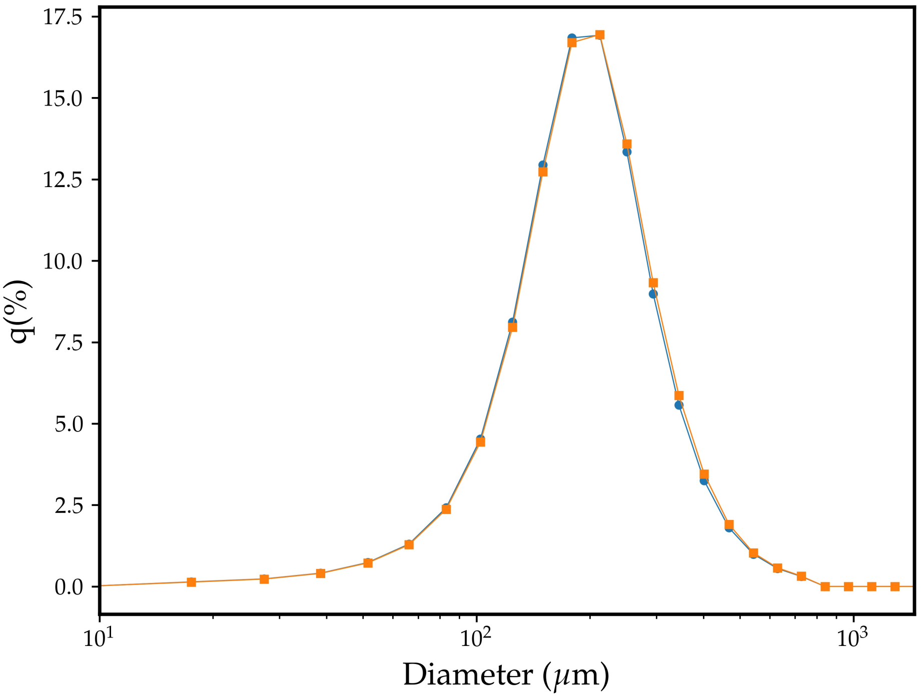

Figure S 4. Particle size distribution for flint waste glass before sintering according to the sieve powder granulometry (Mesh) [100,200). Measurements were performed in duplicate. Mean size $\approx$199.5 μm and geometric standard deviation of 1.5 μm.

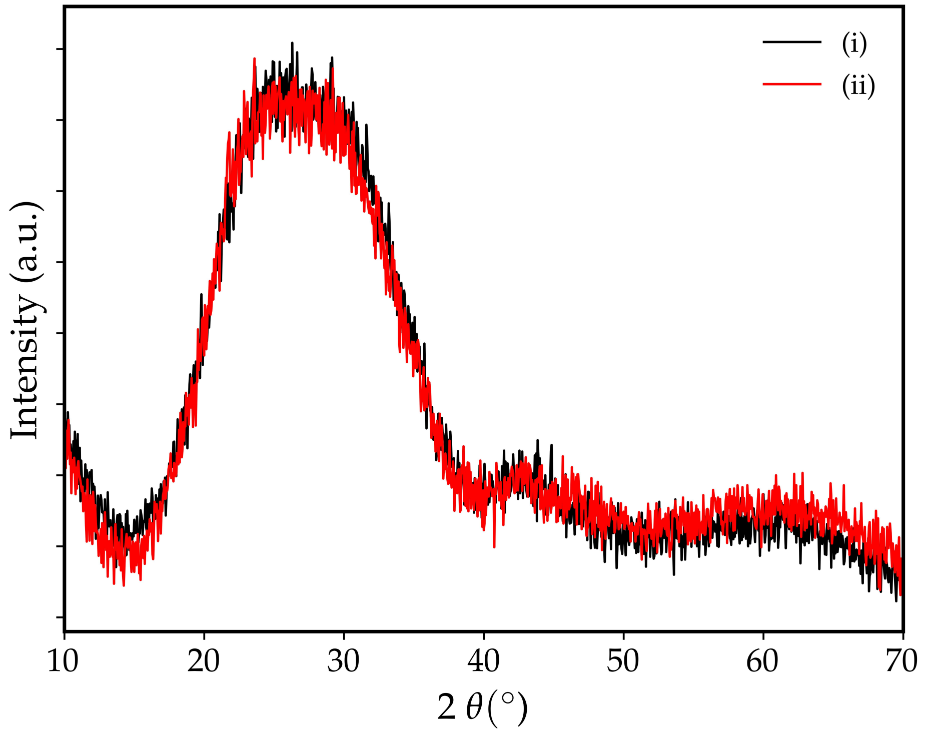

Figure S 5. Powder X-ray diffraction analyses of the silica-based glass without thermal treatment (i) and after thermal treatment (ii), 5 hours at 720 °C. The width of the diffuse halo (approximately 15° 2θ) is consistent with amorphous materials and does not necessarily imply the presence of distinct crystalline phases.
